# Histopathological findings affect quantitative values of single photon emission computed tomography in patients with antiresorptive agent-related osteonecrosis of the jaws

**DOI:** 10.20407/fmj.2022-025

**Published:** 2022-12-27

**Authors:** Taro Okui, Yoshikazu Kobayashi, Madoka Isomura, Masakazu Tsujimoto, Koji Satoh, Hiroshi Toyama

**Affiliations:** 1 Department of Dentistry and Oral-Maxillofacial Surgery, School of Medicine, Fujita Health University, Toyoake, Aichi, Japan; 2 Department of Diagnostic Pathology, School of Medicine, Fujita Health University, Toyoake, Aichi, Japan; 3 Department of Radiology, Fujita Health University Hospital, Toyoake, Aichi, Japan; 4 Department of Radiology, School of Medicine, Fujita Health University, Toyoake, Aichi, Japan

**Keywords:** Bone scintigraphy, Single Photon Emission Computed Tomography/Computed Tomography (SPECT/CT), Antiresorptive Agent-Related Osteonecrosis of the Jaw (ARONJ), Pathological evaluation, Quantitative value

## Abstract

**Objectives:**

This study investigated the relationships between quantitative values calculated from bone single photon emission computed tomography/computed tomography (SPECT/CT) images and histopathological findings observed in surgical specimens from patients with antiresorptive agent-related osteonecrosis of the jaw (ARONJ); it sought to clarify histopathological factors that cause accumulation in bone SPECT/CT images of patients with ARONJ.

**Methods:**

This study included 81 pathological specimens of 21 lesions obtained from 18 patients with ARONJ who underwent SPECT/CT and jaw resection. The maximum standardized uptake value (SUV_max_) of each volume of interest of the specimens was calculated using RAVAT^®^ software. The ratio of the SUV_max_ to the mean value of SUV_max_ in temporal bone was termed rSUV_max_. The rSUV_max_ and pathological findings (sequestration, degree of fibrosis, degree of trabecular bone destruction, degree of inflammatory cell infiltration, and vascularity) were compared using the Mann–Whitney U test and the Kruskal–Wallis test.

**Results:**

In univariate analysis with rSUV_max_ as the dependent variable, the pathological findings of sequestration (P=0.058), degree of fibrosis (P=0.810), degree of trabecular bone destruction (P=0.237), degree of inflammatory cell infiltration (P=0.120), and vascularity (P=0.111) showed no significant difference among the groups for each variable.

**Conclusions:**

We found no association between quantitative values in bone SPECT/CT and histological changes in ARONJ, probably because bone SPECT/CT has limited spatial resolution. Limitations of this study may include the imaging findings of a decrease in tracer accumulation because of an involucrum of necrosed bone, various histopathological findings in ARONJ, and failure to consider the effect of preoperative anti-inflammatory treatment.

## Introduction

Antiresorptive agent-related osteonecrosis of the jaw (ARONJ) is an intractable type of osteonecrosis of the jaw that occurs in patients treated with bone-modifying agents, which are useful in preventing bone-related events in patients with cancer, multiple myeloma, and osteoporosis.^1^ Since the first report of drug-induced osteonecrosis of the jaw by Marx,^2^ numerous reports have been published concerning the treatment and management of ARONJ; thus, ARONJ is an important issue for clinicians. ARONJ is intractable and severely reduces the quality of life in affected patients; the interruption of bone-modifying agent administration is problematic.

Conservative treatment was previously recommended for patients with ARONJ;^1,3,4^ however, several reviews have reported that surgical treatment is superior in this decade.^5–8^ The most difficult aspect of planning ARONJ resection involves determining the boundary between the bone to be resected and the bone to be preserved, then establishing a sufficient area of bone resection, considering the state of inflammation, sequestrum, and blood flow. Incomplete resection can result in lesion recurrence, whereas excessively invasive surgery hinders postoperative prosthetic treatment and may exacerbate cosmetic deficiencies.^9^

The usefulness of various modalities in diagnosing the spread of ARONJ has been widely discussed in the literature.^7–13^ A hybrid single photon emission computed tomography/computed tomography (SPECT/CT) system is currently in use; this system improves the diagnostic accuracy of nuclear medicine images by combining a functional examination (scintigraphy) and a morphological examination (CT). Some researchers have reported the usefulness of SPECT/CT in the diagnosis of ARONJ.^7–13^ SPECT/CT can detect evidence of ARONJ with greater sensitivity than other imaging modalities, such as CT, magnetic resonance imaging, or plain scintigraphy.^13^

Additionally, SPECT/CT has been used for preoperative assessment of ARONJ;^14–16^ the extent of the accumulation range on preoperative SPECT was consistent with the pathological findings in resected specimens. Additionally, the usefulness of SPECT quantitative analysis for ARONJ has been reported in recent years.^17–22^ Notably, the quantitative values of SPECT increase with ARONJ severity^20^ and decrease after anti-inflammatory therapy, such as antibacterial chemotherapy and hyperbaric oxygen therapy.^21^

However, it has been unclear whether pathological changes increase the accumulation of Tc-99m-hydroxymethylene diphosphonate in bone SPECT/CT images of ARONJ. To our knowledge, no studies have compared quantitative SPECT values with pathological findings. Clarification of the relationship between local pathological findings and quantitative values of SPECT in patients with ARONJ could facilitate preoperative assessment of affected patients. Therefore, this study focused on the relationships between quantitative values calculated from SPECT/CT images and detailed histopathological findings in surgical specimens to clarify the histopathological factors that cause accumulation in SPECT/CT images of patients with ARONJ.

## Methods

### Study Patients

We enrolled consecutive 18 patients (eight women and 10 men, median age [interquartile range]: 78 [73.5–80 years]) with 21 lesions (11 mandibles and 10 maxillae) from April 2017 to October 2020. All the patients were initially diagnosed and underwent treatment during this period. The baseline characteristics and numbers of specimens are shown in Table 1. Eight patients had lesions in the mandible (one of these patients had bilateral lesions), eight patients had lesions in the maxilla, and two patients had bimaxillary lesions. The target illnesses for bone-modifying agent administration were malignant diseases (e.g., prostate, breast, and lung cancers) in 11 patients and osteoporosis in seven patients. Of the 21 lesions, four were at stage 1, 11 were at stage 2, and six were at stage 3 of ARONJ. Most lesions were at stage 2; considering the potential for a full cure of the disease, surgical treatment is performed for lesions at stage 1 in our institution. The patients with stage 1 lesions who were cured by conservative therapy such as antimicrobial agents were excluded from the study in advance. The surgeries were segmental mandibulectomy for three lesions, marginal mandibulectomy for eight lesions, and partial maxillary resection for 10 lesions. Segmental mandibulectomy was performed for two lesions at stage 3 and one lesion at stage 2. Patients were diagnosed with ARONJ in accordance with the American Association of Oral and Maxillofacial Surgeons definition.^1^ They underwent bone SPECT/CT scans of the head and neck regions; they also underwent procedures such as high-spatial-resolution CT and magnetic resonance imaging. Subsequently, they underwent jaw resection for treatment of ARONJ. This study was approved by the Ethics Review Committee of Fujita Health University (reference number: HM17-266). Data obtained before September 2017 were retrospectively reviewed without informed consent from patients; these patients were provided an adequate opt-out period (September 2017 to March 2022), and the information was published on our institutional website. Other patients who were prospectively observed received a full explanation of the purpose of the study, then provided written informed consent.

### Bone SPECT/CT procedure

Bone SPECT studies were performed using hybrid SPECT/CT (Symbia T6, Siemens Medical Solutions USA, Hoffman Estates, IL, USA). Data were obtained by SPECT/CT imaging of the head and neck region at 3 hours after intravenous injection of 740 MBq of Tc-99m-hydroxymethylene diphosphonate (Clear Bone Injection, Nihon Medi-physics, Tokyo, Japan). The conditions for acquisition of SPECT images were as follows: matrix, 128×128; pixel size, 3.90×3.90×3.90 mm^3^; slice thickness, 3.90 mm; enlargement, 1.23-fold; main energy window, 140 keV±7.5%; and sub energy window for scatter component estimation, 7% below 140 keV. The number of views was 64 over 360 degrees; the orbit was non-circular in continuous mode. SPECT image reconstruction was performed using a reconstruction algorithm in Flash 3D.^23^ The number of subsets was eight, the number of iterations was 18, and the Gaussian filter was set to 8 mm.^24^ Low-dose CT scans of the head and neck region were performed as follows: non-contrast; slice thickness, 3.0 mm; slice interval, 1.5 mm; tube voltage, 130 kV; tube current, 50 mAs; and matrix, 512×512. B08s SPECTAC was used for the reconstruction function. These images were sent directly to the bundled workstation and manually merged.

**Figure 1:**
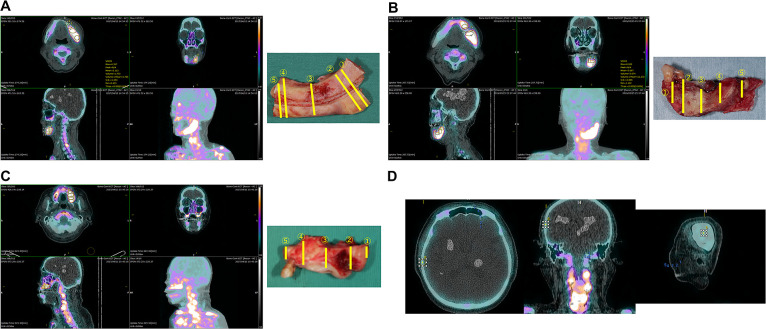
Volume of interest establishment in RAVAT^®^ software A columnar volume of interest (VOI) was manually placed over the corresponding location of each pathological specimen. SUV_max_ values within VOIs were automatically calculated by the software. A. Segmental mandibulectomy from 33 to left mandibular notch in a 78-year-old woman (patient #1). B. Marginal mandibulectomy from 32 to left anterior mandibular ramus in an 80-year-old man (patient #6). C. Partial maxillectomy from 23 to left pterygomaxillary sutures in a 74-year-old man (patient #11). D. Control of the columnar VOI was established over the area without gamma-ray accumulation in the temporal bone.

Image analysis was performed by one oral and maxillofacial surgeon using the closed-source free software RAVAT^®^ version 1.00 (2019, Nippon Medi-Physics, Tokyo, Japan). A columnar volume of interest (VOI) was manually placed over the corresponding location of each pathological specimen in accordance with the following criteria: horizontally, buccal-lingual width diameter of the jaw; vertically, from the alveolar crest to the mandibular canal (in lesions treated by marginal mandibulectomy) or the mandibular inferior border ( in lesions treated by segmental mandibulectomy) or the floor of the maxillary sinus and nasal cavity (in lesions treated by partial maxillectomy) (Figure 1). For quantitative assessment, the standardized uptake value (SUV) was calculated using the following formula.

SUV=SPECT pixel value[countsvoxel]×CCF(BqmLcountvoxel)injected dose [Bq]/weight (g)×100(%)

where CCF is the proportionality coefficient (i.e., cross-calibration factor) between the pixel value and the radioactivity dose calculated from a cylindrical phantom that contains a known amount of radioactivity.^25^

The measurement parameters were the maximum SUV (SUV_max_). Additionally, considering differences in individual bone metabolism, control of the columnar VOI was established over the area without gamma-ray accumulation in the temporal bone; its SUV_max_ was calculated. The ratio of the SUV_max_ to the mean value of SUV_max_ in the temporal bone was termed rSUV_max_. Measurements were performed three times on different days by one oral and maxillofacial surgeon, and the mean value was calculated.

### Determination of resection range

The surgical resection range was determined in a team conference of oral and maxillofacial surgeons using high-accumulation areas on SPECT/CT as the lesion range, morphological changes in cortical and trabecular bone on high-spatial-resolution CT and magnetic resonance imaging, and intraoral findings. The surgical procedures were partial maxillectomy, marginal mandibulectomy, or segmental mandibulectomy.

### Pathological findings

**Figure 2:**
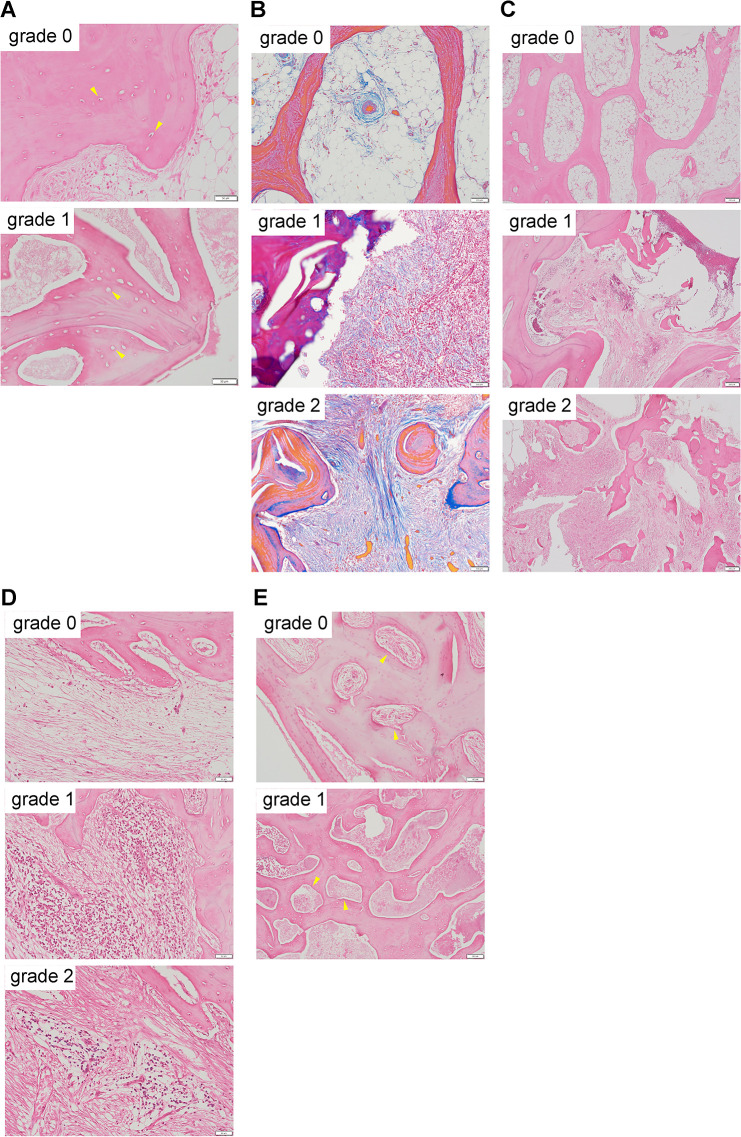
Pathological findings in representative lesions A. Assessment of sequestration (hematoxylin and eosin [H&E] stain, ×100). Top image indicates grade 1 sequestration: osteocytes are present in bone lacunae (arrowhead). Bottom image indicates grade 2: osteocytes are not present in bone lacunae (arrowhead). B. Assessment of tissue fibrosis in the bone marrow space (Azan stain, ×200). Top image indicates grade 0: no or very mild fibrosis. Middle image indicates grade 1: moderate fibrosis. Bottom image indicates grade 2: severe fibrosis. C. Assessment of trabecular bone destruction (H&E stain, ×40). Top image indicates grade 0: no or very mild trabecular bone destruction. Middle image indicates grade 1: moderate trabecular bone destruction. Bottom image indicates grade 2: severe trabecular bone destruction. Middle and bottom images show destruction of the trabecular bone structure because of the invasion of granulation tissue and fibroblasts. D. Assessment of inflammatory cell infiltration (H&E stain, ×200). Various degrees of chronic and acute inflammatory cell infiltration are present in the bone marrow space. Top image indicates grade 0: mild inflammatory cell infiltration. Middle image indicates grade 1: moderate inflammatory cell infiltration. Bottom image indicates grade 2: severe inflammatory cell infiltration. E. Assessment of vascularity in the bone marrow (H&E stain, ×100). Top image indicates grade 0: endothelial cells are present in the blood vessel (arrowhead). Bottom image indicates grade 1: endothelial cells are not present in the blood vessel (arrowhead), and the vessel is filled with a bacterial mass.

Specimens used for clinical diagnosis were reevaluated for this study. Surgical specimens were sliced at approximately one tooth interval, then stained with hematoxylin and eosin, as well as the Azan staining method. One oral and maxillofacial surgeon and one oral pathologist evaluated each specimen on the basis of the following factors: sequestration, defined as the presence or absence of osteocytes in bone lacunae (grade 0 or 1); degree of tissue fibrosis in the bone marrow space (grade 0–2); degree of trabecular bone destruction (grade 0–2); degree of inflammatory cell infiltration (grade 0–2); and vascularity, defined as the presence or absence of vascular endothelial cells in blood vessels (grade 0 or 1) (Figure 2). When different pathological assessments were observed in a single specimen, the grade that covered the larger area was adopted.

### Statistical analysis

Univariate analysis was performed, with rSUV_max_ as an objective variable and each pathological finding as an explanatory variable. The Mann–Whitney U test was used for comparisons between two groups, whereas the Kruskal–Wallis test was used for comparisons among ≥3 groups. Differences were considered statistically significant at P<0.05. Statistical analyses were performed using EZR ver. 1.38 (available at http://www.jichi.ac.jp/saitama-sct/SaitamaHP.files/statmedEN.html).

## Results

**Figure 3:**
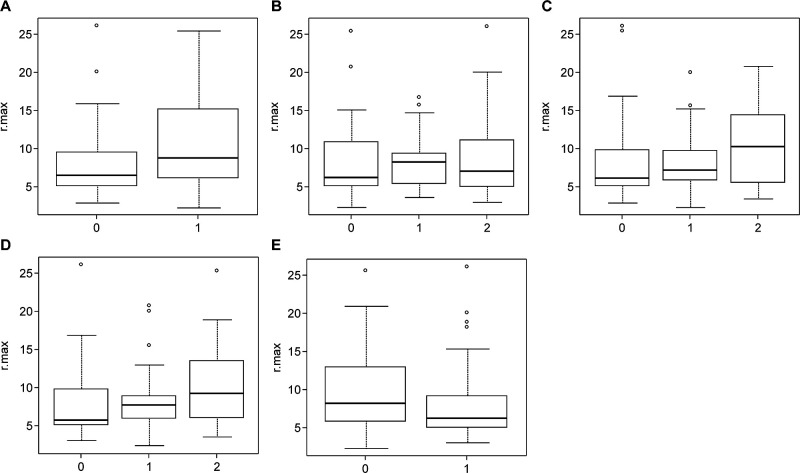
rSUV_max_ values for each pathological finding A. Sequestration B. Degree of fibrosis C. Degree of trabecular bone destruction D. Degree of inflammatory cell infiltration E. Vascularity Pathological findings did not significantly differ according to rSUV_max_. rSUV_max_, ratios of maximum standardized uptake values.

This study included 81 pathological specimens of 21 lesions from 18 patients. The univariate distribution of rSUV_max_ with respect to each pathological finding is shown in Figure 3. Concerning sequestration, median [interquartile range], (n) values for rSUV_max_ were grade 0, 6.57 [5.17–9.56], (55) and grade 1, 8.91 [6.35–14.72], (26); differences between grades were not statistically significant (P=0.058). Regarding the degree of fibrosis, values for rSUV_max_ were grade 0, 6.26 [2.29–10.87], (19); grade 1, 8.28 [5.48–9.43], (19); and grade 2, 7.16 [4.99–11.21], (43). Differences among these grades were not statistically significant (P=0.810). With respect to the degree of trabecular bone destruction, values for rSUV_max_ were grade 0, 6.08 [5.12–9.89], (32); grade 1, 7.16 [5.91–9.75], (33); and grade 2, 10.25 [5.92–13.92], (16). Differences among these grades were not statistically significant (P=0.237). In terms of inflammatory cell infiltration, values for rSUV_max_ were grade 0, 5.68 [5.02–9.78], (24); grade 1, 7.71 [5.89–8.93], (29); and grade 2, 9.17 [6.16–13.51], (28). Differences among these grades were not statistically significant (P=0.120). Concerning vascularity, rSUV_max_ were grade 0, 8.21 [6.01–12.92], (38) and grade 1, 6.26 [5.03–9.17], (43); differences between grades were not statistically significant (P=0.111). No pathological evaluation items significantly differed according to rSUV_max_.

## Discussion

When planning the surgical treatment of ARONJ, it is difficult to clarify the boundary between the bone to be resected and the bone to be preserved, then establish the necessary and sufficient resection range based on bone vitality, bone inflammation/infection status, and bone structural changes.^26,27^ Bone scintigraphy can detect bone metabolic activity with high sensitivity before structural changes occur,^28^ and SPECT/CT can be combined with CT to accurately localize the lesion.^13,14^ Some studies have indicated that the preoperative range of bone SPECT/CT accumulation is correlated with the extent of lesions in actual surgical specimens.^14,16,29^ The resection range was determined through preoperative assessment of the accumulation range of bone SPECT. The resection was considered sufficient because no recurrence was observed in any lesions. However, overtreatment could not be ruled out.

This study did not find significant associations between SUV values obtained from bone SPECT/CT and the results of histopathological analysis. However, the rSUV_max_ tended to increase with lesion progression in terms of sequestration, bone destruction, inflammatory cell infiltration, and vascularity. In areas where sequestration or bone destruction advanced because of ARONJ progression, bone hypermetabolism may have occurred. The bone SPECT/CT radiotracer Tc-99m-hydroxymethylene diphosphonate is highly concentrated in areas of increased bone metabolism.^24^ Therefore, our results are reasonable. However, our grading system did not reveal significant differences. Although inflammatory cell infiltration is not an indicator of bone condition, the rSUV_max_ tended to increase with the degree of inflammation, suggesting that radiotracer accumulation increases because of increased bone metabolism as a defense response against bacteria that adhere to bone; moreover, the degree of inflammatory cell infiltration increases during the same process. The degree of inflammatory cell infiltration is presumed to be easily influenced by anti-inflammatory treatments, such as antibacterial agents and hyperbaric oxygen therapy; rSUV_max_ values are reportedly affected by anti-inflammatory treatments.^21^ In terms of vascularity-specific histopathological findings, vascular destruction (i.e., loss of vascular endothelial cells in blood vessels) occurs in the presence of increased inflammation. A process of decreased blood flow-dependent accumulation of radiotracer and decreased rSUV_max_ may be involved.

The following factors may have contributed to the unexpected results. First, a technical factor could be the limited spatial resolution of bone scanning from the scattered radiotracer activity. The small amount of necrotic bone may be masked by high accumulation in the surrounding area, thus hindering identification of a high-accumulation area.^14,29^ In a comparative study involving qualitative assessments of bone SPECT/CT accumulation intensity and histopathological findings, Miyashita et al. found no difference in bone SPECT/CT accumulation intensity between the hypermetabolic area near the necrotic bone and the surrounding hypermetabolic area.^15^

Second, the so-called “cold-in-hot” effect may influence the contradiction between the advancement of bone necrosis and the reduction of tracer accumulation. This effect is generally observed around an involucrum of necrosed bone. Sequestrum bone separation occurs with disease progression and loss of blood flow; thus, the tracer remains in the surrounding area, and a “hot area” is observed around the “cold area.”^13,20^

Third, there is a wide variety of histopathological findings in ARONJ lesions. The pathophysiology of ARONJ has not been completely elucidated; it involves uneven morphological changes in bone, which consist of necrotic bone and regenerative tissue in various inflammatory processes from acute to chronic.^26^ We observed lesions with different histopathological findings above and below the mandibular canal. Here, we assessed specimens using a single pathological evaluation approach, although there were multiple degrees of morphological changes. In a study that evaluated ARONJ using fluorodeoxyglucose-positron emission tomography/CT, marginal resection was considered indicative of poor prognosis when accumulation had progressed to the lower part of the mandibular canal.^30^ During the excision of mandibular lesions, there are substantial differences in terms of surgical invasiveness, such as the requirement for reconstruction and the preservation of postoperative quality of life that must be balanced between marginal resection (maintains mandibular continuity) and segmental resection (disrupts mandibular continuity). In future studies, the VOI should be separately established both above and below the mandibular canal on bone SPECT/CT images used to measure the SUV.

Fourth, the extent and intensity of bone SPECT/CT accumulation are reduced by anti-inflammatory treatments, such as antibacterial agents and hyperbaric oxygen therapy.^21^ In the present study, we did not examine the effects of anti-inflammatory treatment between the date of bone SPECT/CT imaging and the date of surgery. There was a median interval of 8.9 weeks [interquartile range: 5.6–10.6] between bone SPECT/CT imaging and surgical treatment. For 11 lesions, oral administration of penicillin antibiotics was performed at the discretion of the treating physician. Therefore, we cannot rule out a discrepancy concerning the degree of inflammatory cell infiltration between preoperative bone SPECT/CT quantification and post-resection histopathological findings. Otherwise, ARONJ may have progressed during the waiting period of approximately 2 months. Thus, it was difficult to characterize the relationship between the SUV of the VOI and the histopathological findings of the resected specimen.

Importantly, this study did not demonstrate histopathological validation of the appropriate resection range based on the accumulation of bone SPECT/CT. Excessive resection exacerbates postoperative dysfunction. There is a need to determine whether quantitative values (e.g., SUV obtained from bone SPECT/CT) can serve as objective indicators for identification of the resection boundary; these values should be evaluated at the resection margin. Miyashita et al.^15^ reported that resection based on bone SPECT/CT accumulation is a broad surgical approach that involves the removal of necrotic bone from the necrotic core to vascular viable margins. In the present study, VOIs were not established inside and outside of the resection margin, and the cutoff values of quantitative assessments were not tested in terms of resection. These are important considerations that should be addressed in future studies.

Quantitative values in bone SPECT/CT are reportedly correlated with degrees of disease exacerbation,^20^ as well as changes in individuals before and after anti-inflammatory treatment.^21^ However, our findings suggest that quantitative values in bone SPECT/CT may not be readily applicable for evaluation of local pathological changes. The relationships between histopathological findings in patients with ARONJ and the quantitative values in bone SPECT/CT should be clarified using a more rigorous study design and larger number of cases. The usefulness of bone SPECT/CT will increase if its quantitative values can be used to plan surgical treatment of ARONJ.

In conclusion, we collected high spatial resolution bone SPECT/CT images, subdivided the lesion in each image, established multiple VOIs within each lesion, and measured the SUV that indicated the intensity of tracer accumulation in the lesion. Furthermore, by comparing histopathological findings in the areas that matched the VOIs, we explore the use of quantitative values of accumulation intensity as objective indicators to determine the resection range for ARONJ lesions; however, the results were not as expected.

The quantitative values in bone SPECT/CT suggested that it is difficult to evaluate pathological changes according to location, such as the boundary between normal and diseased areas within a single lesion. However, there is a need to consider the establishment of more rigorous criteria for preparing pathological specimens and comparing them with SUVs.

## Figures and Tables

**Table1:** Patient characteristics and numbers of specimens

Patient	Lesion	Age (years)	Sex	Maxilla/Mandible	Stage	Surgical procedure	Target illness	Number of slides
1	1	78	F	Mandible	2	Segmental mandibulectomy	Osteoporosis	5
2	2	84	M	Mandible	3	Segmental mandibulectomy	Prostate cancer	5
3	3	74	M	Mandible	2	Marginal mandibulectomy	Lung cancer	4
4	4	87	M	Mandible	2	Marginal mandibulectomy	Prostate cancer	4
5	5	73	M	Mandible	3	Segmental mandibulectomy	Prostate cancer	2
	6			Mandible	1	Marginal mandibulectomy		5
6	7	80	M	Mandible	2	Marginal mandibulectomy	Prostate cancer	5
7	8	78	F	Mandible	2	Marginal mandibulectomy	Osteoporosis	5
8	9	79	M	Mandible	1	Marginal mandibulectomy	Prostate cancer	3
9	10	75	F	Mandible	2	Marginal mandibulectomy	Osteoporosis	4
	11			Maxilla	3	Partial maxillectomy		3
10	12	79	F	Mandible	1	Marginal mandibulectomy	Osteoporosis	2
	13			Maxilla	2	Partial maxillectomy		3
11	14	74	M	Maxilla	3	Partial maxillectomy	Prostate cancer	5
12	15	80	F	Maxilla	2	Partial maxillectomy	Osteoporosis	5
13	16	71	M	Maxilla	2	Partial maxillectomy	Prostate cancer	4
14	17	72	M	Maxilla	3	Partial maxillectomy	Prostate cancer	4
15	18	47	F	Maxilla	3	Partial maxillectomy	Breast cancer	3
16	19	63	F	Maxilla	2	Partial maxillectomy	Breast cancer	4
17	20	83	F	Maxilla	2	Partial maxillectomy	Osteoporosis	3
18	21	83	M	Maxilla	1	Partial maxillectomy	Osteoporosis	3
